# Two Interesting Parasitic Diseases

**Published:** 1869-07-01

**Authors:** 


### *Two Interesting Parasitic Diseases.*

J. H. Salisbury, M.D., Cleveland, Ohio, (*Boston Medical and Surgical Journal*), mentions two interesting parasitic diseases, with their treatment.

1st. *Chloasma produced by the Microsporon furfur* (Robin).—A cigar-maker, aged 30, came to him in January last, covered over the whole trunk, anteriorily and posteriorily, with brownish yellow maculæ, or spots of irregular outline, from the size of a pin-head to four and six lines in diameter. The spots were not elevated above what appeared to be the surrounding skin. The epidermis was soft and pultaceous, and appeared spongy when scraped with a scalpel. The colored cuticle peeled off like the epidermis from a boiled apple. The sheets and night-dresses were covered with furfuraceous scales. On microscopical examination, the epidermis was found filled with a fungus (*Microsporon furfur*), both in the spore and filamentous stages of development. The spores were developed in multitudes in the dark spots.

*Treatment.*—The patient was pale and emaciated, and was disturbed at night by alternate sweating and chilliness. A diet of rare beef and bread was ordered, and he was put on the following treatment:

℞. Acid, sulphuric aromat. ℥ iij.—S. Put two teaspoonfuls in half a pint of warm water, and wash the body and limbs all over every other night, and wipe dry afterwards.

℞. Nichols' sol. bisulphite of soda, ℥ iij.—S.

Put $1\frac{1}{2}$ tablespoonfuls in half a pint of warm water, and wash the body and limbs all over every other night, and wipe dry. ℞ Tr. ferri chlorid. ℥ iij.—S. Take twenty drops in a full glass of water two hours after each meal. ℞ Tr. cinchonæ comp. ℥ vj.; potass. bromid. ʒ iv.—S. Take a teaspoonful before each meal.

On the fourth day of treatment the spots had entirely disappeared. Sickly spores and filaments remained in the epidermis, but further development seemed to be checked. Treatment was continued. At the end of two weeks, the vegetation disappeared, the skin was smooth and healthy, and the patient had gained several pounds in weight, and was feeling perfectly well.

2d. *Parasitic Disease of the Conjunctival Membrane and Epidermis of Cheek.*—A carpenter, aged 26, came to him with a diffuse inflammation, roughness and ædema of the eyelids and the surrounding soft parts. The inflammation and scaly condition extended over nearly the whole cheek, to the wing of the nose, and from this point, up the ridge of this organ, to the forehead, involving the entire eyebrow. At first it was considered a case of erysipelas, but on examination, the epidermis was filled with a fungoid growth, in the spore and filament stages of development. This vegetation extended to the conjunctival membrane lining the eyelids. The following was prescribed: ℞ Dilute citrine ointment, ℥ ij., glycerine, ʒiij.—M.S. Apply morning, noon and night, to the inside of eye and over the entire affected parts. ℞ Nichols' sol. bisulphite of soda, ʒ ss.; aquæ, ℥ viij.—M. With an atomizer, the spray of this mixture was thrown into the eye and over the cheek, for five minutes, morning and evening, after washing and before applying the ointment. ℞ Tr. ferri. chlorid. ʒiij.—S. Take twenty drops in a full tumbler of water two hours after breakfast and dinner. The use of sweets and all organic acids was forbidden, and a plain, substantial diet ordered. The patient rapidly recovered, and in three weeks there was not a trace of the parasitic growth.

